# Reference Electrodes with Polymer-Based Membranes—Comprehensive Performance Characteristics

**DOI:** 10.3390/membranes9120161

**Published:** 2019-11-29

**Authors:** Peter Lingenfelter, Bartosz Bartoszewicz, Jan Migdalski, Tomasz Sokalski, Mirosław M. Bućko, Robert Filipek, Andrzej Lewenstam

**Affiliations:** 1 Center for Process Analytical Chemistry and Sensor Technology (ProSens), Åbo Akademi University, Piispankatu 8, FI-20500 Turku, Finland; peter.lingenfleter@gmail.com (P.L.); tsokalsk@abo.fi (T.S.); 2 Faculty of Materials Science and Ceramics, AGH-University of Science and Technology, Al. Mickiewicza 30, PL-30059 Cracow, Poland; bartoszbartosz92@o2.pl (B.B.); migdal@agh.edu.pl (J.M.); bucko@agh.edu.pl (M.M.B.); rof@agh.edu.pl (R.F.)

**Keywords:** potentiometry, reference electrode, solid contact, heterogenous membranes, polymer membranes

## Abstract

Several types of liquid membrane and solid-state reference electrodes based on different plastics were fabricated. In the membranes studied, equitransferent organic (QB) and inorganic salts (KCl) are dispersed in polyvinyl chloride (PVC), polyurethane (PU), urea-formaldehyde resin (UF), polyvinyl acetate (PVA), as well as remelted KCl in order to show the matrix impact on the reference membranes’ behavior. The comparison of potentiometic performance was made using specially designed standardized testing protocols. A problem in the reference electrode research and literature has been a lack of standardized testing, which leads to difficulties in comparing different types, qualities, and properties of reference electrodes. Herein, several protocols were developed to test the electrodes’ performance with respect to stability over time, pH sensitivity, ionic strength, and various ionic species. All of the prepared reference electrodes performed well in at least some respect and would be suitable for certain applications as described in the text. Most of the reference types, however, demonstrated some weakness that had not been previously highlighted in the literature, due in large part to the lack of exhaustive and/or consistent testing protocols.

## 1. Introduction

Potentiometry, applied routinely in environmental measurements, process analysis, and extensively in clinical chemistry, regained research interest owing to revolutionary developments in electrode design and new materials used. An evident boost to the development activity came with the invention of solid-contact electrodes, which paved the way to all-solid-state electrodes. These electrodes are fully integrated, which allows for different architectures, miniaturization, favorable electrochemical and metrological properties, sterilization, and mass production by unified techniques, such as injection molding or 3D printing [1]. 

Along with indicator electrodes, their partners in galvanic cells, reference electrodes were developed. As in the case of ion-sensors this was achieved by using ion-to-electron transducing layers, e.g., conducting polymers (CPs) [2].

The reference electrode (RE) is an indispensable and crucial component in potentiometry and open-circuit sensor technology as well as a reference point in amperometric measurements. The failure of the reference electrode means the failure of the entire system. Thus, the quality of the reference electrode is critical in electrochemical measurements, and especially those where multi-parameter analyses are performed. Furthermore, most of the lifetime and size reduction gains from the indicator electrode optimization with CPs are superfluous if the reference electrode cannot be miniaturized in an identical manner.

The classical method of making a reference electrode is by using an electrode of the second kind (Ag/AgCl or Hg/Hg_2_Cl_2_) in contact with a solution of a chloride electrolyte, so that the potential determining process is fast and reversible, while the potential itself is stable and reproducible over time, because the composition of the electrolyte is maintained constant. The latter is achieved by placing the electrode in a compartment separate from the sample. This separation, however, allows some electrolytic contact via the salt bridge. To minimize the diffusion potential generated at this junction, the bridge is filled with an equitransferent electrolyte, typically KCl, at a high concentration. Therefore, also in the electrode compartment, the chloride electrolyte is often KCl. A redox electrode (of the 0^th^ kind) in a solution containing a redox couple is sometimes used instead of a RE of the second kind. In principle, this approach is the same as described above.

The quality of the reference electrode is especially important in the direct potentiometric measurement of pH and blood electrolytes where the potential of several indicating electrodes is measured, often in a high-throughput and minimal sample volume automatic analyzer, and where the sample concentrations are measured over a broad range (pH), or in the range where junction potentials are particularly variable (blood electrolytes). In zero-current potentiometry, it is expected that a reference electrode supports reliable measurements. In other words, it is expected that it is sufficiently stable, that it is not fouled by the samples, and that the reference electrode itself does not contaminate the samples. Ideally, it is expected that a reference electrode is easy to manufacture and use, service-free, cheap, and robust. 

Although this would seem fairly easy to accomplish, the large body of literature on the subject would argue otherwise [3,4,5,6,7,8,9,10,11,12,13,14,15,16,17,18,19,20]. The nature of the liquid junction at the salt-bridge plays an especially critical role. When, for instance, the liquid junction becomes clogged, or if the liquid junction is poorly manufactured, errors arise from substantial liquid-junction potentials, which vary with the ionic composition of the solution under test. Optimally, the reference electrode would be of the free-diffusion type or ideally junctionless. The reference junction becomes more and more critical when the ionic strength of the liquid under test is very high or very low, as with, e.g., natural waters and especially power plant water. It has furthermore been shown that calculated residual liquid junctions for these types of samples do not always match what is experimentally exhibited. The contribution of the liquid junction potential arising at the filling solution/sample interface requires often a firm comprehension of potentiometry and possible correction [3,19,20]. 

Furthermore, miniaturization of liquid-junction-type conventional reference electrodes is difficult, due to the requirement of regular maintenance and a vertical working position. Attempts have been made to develop small REs using the classical approach [21]. The electrolyte (KCl) was placed in a porous material deposited on a flat Ag/AgCl pellet as the substrate. On top of this “immobilized” KCl, the authors placed a film, also porous but with much smaller pores to slow down the release of KCl from the electrode and the contamination of the internal layer with the species from the sample. Obviously, the failure in this approach is lifetime: the smaller the electrode, the faster it is depleted of KCl and contaminated by the sample. Indeed, the authors of [21] reported a 20–90-min lifetime. 

Of practical relevance, there are other approaches to the reference electrode, in particular those not utilizing a liquid junction. Since most potentiometric measurements for analytical and thermodynamic purposes are made using cells with liquid junctions, the diffusion potential is observed. A diffusion potential occurs at the boundary between two electrolytes of different composition, where there is a concentration gradient, and thus ion diffusion takes place. Due to different ionic mobilities, some of the ions move faster than others. The different diffusion flows lead to charge separation, and thus an electric field is established. The electric field holds the fast-moving ions and accelerates the slower ions. In the end, a steady state is attained in which equal amounts of the involved ions are transported by a combination of migration and diffusion. Using standard thermodynamic relationships and integrating over the entire boundary region for all species, it can be shown that [22,23]:

(1) $$E_{D}=\frac{-RT}{F}\int_{A}^{B}\sum_{i}\frac{t_{i}}{z_{i}}{\;d\;ln\;a}_{i}$$

where *E_D_* is the diffusion potential, *A* and *B* denote two solutions with different compositions, and *t_i_*, *z_i_*, and *a_i_* are the transference number, charge, and activity of the *i*^th^ species, respectively, *R* is the gas constant, *T* is the temperature, and *F* is the Faraday constant. It must be noted that this equation is valid regardless of the physical nature of the liquid junction, but, as it involves single ion activities, it cannot be evaluated purely within the framework of thermodynamics. In order to integrate this equation, non-thermodynamic assumptions must be made. The simplest approach mathematically is to assume linear concentration gradients across the liquid junction and constant activity coefficients and ionic mobilities, which results in the well-known Henderson equation [23,24,25]:

(2) $$E_{D}=\frac{-RT\sum_{i}u_{i}{(c}_{B}{-c}_{A})}{F\sum_{i}u_{i}z_{i}{(c}_{B}{-c}_{A})}ln\frac{\sum_{i}u_{i}z_{i}c_{B}}{\sum_{i}u_{i}z_{i}c_{A}}$$

where, *A* and *B* denote two solutions with different composition, *u_i_* is the mobility, *c_i_* is the concentration, *z_i_* is the charge, *R* is the gas constant, *T* is the temperature, and *F* is the Faraday constant. Various alternative equations have been derived over the years, but in general, although they may be regarded as more rigorous, no significant differences in calculated diffusion potentials can be expected in most cases.

In direct potentiometry, even small changes in the junction potential can lead to erroneous results, especially when the analytical potential range is narrow. One example is human serum, where the usual concentration ranges for ionized calcium, potassium, and sodium are normally 1.0–1.5, 3.0–6.0, and 120–160 mmol/L, which respectively corresponds to potential intervals of 5.2, 17.8, and 6.4 mV [19]. Furthermore, using Henderson’s equation, we can only estimate the diffusion potential. In some cases, this estimation is good enough but in others (human serum) it is questionable. Thus, a few criteria can be formulated with which to evaluate a reference electrode:

(1) The absolute value of the diffusion potential at the liquid junction should be as small as possible.
(2) Changes to the diffusion potential resulting from changes in the sample composition should be as small as possible.
(3) Changes to the diffusion potential resulting from changes to the reference electrode composition should be as small as possible.

These aims are usually achieved by using a high concentration of electrolyte in the bridge of the reference electrode such as 3 M KCl. Such bridges, called hypertonic in biological research, have an intrinsic disadvantage, which is the possibility of denaturation of proteins and/or crystallization of the electrolyte itself. Both may cause instability in the reference electrode potential. The problem may be avoided by using dilute solution in the bridge (isotonic bridge). However, this leads to higher diffusion potentials since isotonic bridges are more sensitive to changes in the sample ionic strength. All of the requirements described make it challenging to build an ideal reference electrode. For the same reason, the number of research papers on reference electrodes is disproportionately small compared to the number of papers on indicator electrodes.

In the past 25 years reference electrode research has begun to regain serious attention [26,27,28,29,30,31,32,33,34,35,36,37,38,39,40,41,42,43,44,45,46,47,48,49,50,51,52,53,54,55,56,57,58,59,60,61,62,63,64,65,66,67,68,69,70]. 

Beyond the conventional designs prevalent worldwide, several other compelling approaches have been explored:

i. Equitransferent Salts Dispersed in a Polymer or Other Solid

The challenge of designing functional solid contact reference electrodes was undertaken by Russel who offered a solid-state membrane for reference electrodes made of a polyvinyl resin doped with a very large amount of KCl (1:1 w/w KCl/resin) fabricated under the commercial name, REFEX. The electrochemical characterization of this material was delivered in 1994 [26,27,28]. Surprisingly, despite the heavy salt loading and large surface area in contact with the liquid sample, the reported leakage of KCl into the sample solution is less than what occurs with conventional ceramic frit junctions. The junction potential is quick to stabilize and relatively constant with time even in media with a very low ionic strength. There are also a number of other papers presenting similar constructions using different polymers or resins, e.g., pressed Al_2_O_3_-PTFE, urea-formaldehyde, poly(methyl methacrylate)—propylene carbonate, and/or polyester resin [29,30,31,32,33]. An all-solid reference electrode consisting of a sintered Ag/AgCl mixture embedded in solid remelted KCl was as well proposed [34]. Although these concepts are rather different on the surface, the unifying factor is the controlled release of equitransferent salt from either a polymer matrix, dense glass, or ceramic sinter. Variations of all-solid-state reference electrodes with polycrystalline powders of tungsten-substituted alkali molybdenum bronzes mixed with polyester resin were offered [33,34,35]. These reference electrodes showed no response to changing pH, Na^+^ concentration or redox potential. Unfortunately, all of these electrodes showed a relatively high electrical resistance (about 1–500 MΩ), and it was reportedly not possible to get reproducible results. 

Recently, the pioneering idea of Russel was extended in the research of Lewenstam’s group [70]. Granholm et al. showed that the REs can be produced by dispersing KCl in polypropylene during injection-molding [71], while Mousavi el al. [72] demonstrated that the polymer of a solid-state reference electrode can serve as an embodiment for ion sensors. 

Furthermore, it was demonstrated that the PVC heterogenous membranes with silver bromide-KBr salts work superbly as the all-solid-state reference electrodes [73] and that both KBr- and KCl-containing reference membranes are excellent internal solid contact for ion-selective electrodes [74].

These reports provide the signal of a breakthrough in the reference electrode technology which may be called “heterogenous membrane revolution” [73,74]. Material-wise, two aspects of novelty are striking: (1) the application of inorganic salts, and (2) new inert binders which constitute a composite membrane and mechanically processable membranes suitable for electrochemical measurements.

ii. Two Ion-Selective Electrode (ISE) Membranes Connected in Parallel

This concept, while illustrative, is only useful in extremely rare cases and has been thoroughly discussed elsewhere [38,39].

iii. Compensated Cationic and Anionic Response in a Polymer Membrane or Conducting Polymer Film owing to Close-to-Equal Permeability

Ionic liquids [44,45,46,47,48,49], quaternary ammonium borates [42,50,51,52,53,54,60,61], or other materials [51,52,55,56] are dispersed in, e.g., polyurethane [21,41,55,56,57,58], poly(vinyl chloride) (PVC) [42,50,51,60,61], poly(vinyl chloride) carboxylated polymer [21], or polyacrylate [52,53,54]. Mediating layers such as Nafion are also sometimes applied [40,41,58]. These also variably make use of a conventional inner solution or solid-contact internal materials consisting of conducting polymers, and/or KCl, or NaCl saturated in water, agar, PVC, silicone rubber, mixtures thereof, or other matrices. 

An RE with 1-dodecyl-3-methylimidazolium chloride ionic liquid as membrane electrolyte was described in [44]. In fact, this electrode is actually a quasi-reference electrode (QRE) since it works only if the sample contains a high sulfate background. This is not surprising, as it has previously been reported that a similar ionic liquid, 1-butyl-3-methylimidazolium hexafluorophosphate, had been developed into a sulfate ion-selective electrode [62].

Further improvement of many, if not most, of these reference electrodes requires a more detailed knowledge of the functional mechanism. For instance, if the mechanism relies on the distribution potential or on the release of QB from membrane to sample, the use of a solid contact construction instead of the conventional setup with internal filling solution is possible. If, however, the electrodes work due to the release of an inorganic electrolyte (e.g., KCl) extracted from the internal filling matrix (be it liquid or solid), further miniaturization is questionable. It seems unlikely that the latter mechanism would be prevalent, as there have been reports of solid-contact reference electrodes (SCREs) lasting for an extended period of up to two years [21]. However, the stability of one type of SCRE cannot preclude another type of SCRE having another functional mechanism altogether, and therefore each type must in the end be considered separately unless certain general aspects allow for the assumption of a universal functional mechanism.

iv. Polyion-Sensitive ISEs Used as Reference Electrodes

This approach extends interesting concepts introduced with the advent of potentiometric membrane electrodes that are responsive to polyionic analytes [63,64]. The main principle is quite simple: if a membrane electrode can be made responsive to a highly charged analyte, the resulting Nernstian response function will exhibit a very small electrode slope that is inversely proportional to the charge of the analyte. If some amount of this analyte is continuously present at the membrane surface, the resulting potential will be nearly independent of its concentration. Since some well-established anticoagulants (such as heparin) are polyions, and membrane electrodes have been specifically designed to measure such anticoagulants in blood, it seems possible to design reference electrode membranes for use in blood samples. The primary disadvantage of this concept is that highly lipohilic ions may ion-exchange with the polyion in the membrane, thereby increasing its response to small ions.

v. Modified Conducting Polymer Reference Electrodes

This classification comprises pH-buffered, multi-layer and overoxidized junctionless reference electrodes with only electrochemically deposited conducting polymers on the conducting substrate [50,65,66,67,68]. A more versatile solution was proposed in [69]. The electrode described contained a layer of a conducting polymer (PEDOT or PMPy) doped with a high concentration of pH buffer. The electrode, in fact, was pH-sensitive, but the sample pH in the vicinity of the electrode was buffered by the electrode itself, thus ensuring a constant potential regardless of the composition of the sample.

Our goal in this work was not to take one variety and improve upon it with the exclusion of all others. Questions of mechanism, miniaturization, etc. are avoided. In this report, we attempt to reproduce and compare electrodes at least similar to those described in [26,27,28,30,35,55,56,57,58,60,61], or as we designated them, REFEX (PVA), urea-formaldehyde (UF), remelted KCl (RKCl), and PVC-(QB(PVC)), and polyurethane-based (QB(PU)) conventional membrane electrodes, where the membranes were loaded with the lipophilic salt, tetrabutyl ammonium tetrabutyl borate (QB). 

## 2. Materials and Methods

For the primary reference electrode, against which all other references were tested, we used a Thermo Orion Ross Ultra double-junction reference electrode purchased from Thermo Fisher Scientific, Waltham, MA, USA. The behaviors of the electrodes were compared with two commercial electrodes: an Orion Ross Sureflow double-junction reference electrode purchased from Thermo Fisher Scientific, Waltham, Massachusetts, USA and a REFEX reference electrode obtained from Refex Sensors Ltd, Westport, C. Mayo, Ireland.

### 2.1. Chemicals and Materials

Aqueous standard and test solutions were prepared from analytical grade reagents and Elga deionized water (18.2 MΩ·cm). Selectophore® grade high molecular weight poly(vinyl chloride) (PVC), and tetrahydrofuran (THF) were purchased from Sigma Aldrich (Steinheim, Germany). Tetrabutylammonium tetrabutylborate (97%, QB), 2,2-dimethoxy-2-phenylacetophenone (DMPP), vinyl acetate (≥99.0%, VA), poly(vinyl acetate) (M_w_ ~100,000), silver wire (≥99.9%), potassium chloride (≥99.0%), formaldehyde solution (purum, 37% in water stabilized with 10% methanol), urea (≥99.5%), sodium acetate (≥99%), and DIN19266 pH 4.008, 6.865, 7.413, and 9.180 buffer standard solutions were purchased from Sigma Aldrich (Steinheim, Germany). Tecothane® polyurethane (PU) was obtained from Lubrizol. Air release additive BYK®-A 515 was obtained from Algol Chemicals Oy (Espoo, Finland). 

### 2.2. Preparations

i. Preparation of Basic Cocktails for Membranes

For the purpose of this report, the term “basic cocktail” refers to solutions of PVC or PU, plasticizer, and organic electrolyte (if applicable) in THF. These cocktails are fully transparent homogeneous solutions.

Basic cocktails were prepared as follows: appropriate amounts of organic electrolyte (QB) [60,61] were placed into 4 ml sample vials. The required exact weights of other components were calculated in accordance with the actual weight of the organic electrolyte and then added. The vials were shaken to mix the ingredients and then THF was added. The vials were allowed to spin on a Stuart SRT6 roller mixer overnight to ensure complete dissolution of the PVC or PU. An ultrasound was used when the PVC proved difficult to dissolve. A 15% dry mass was used with the PVC basic cocktails and an 8% dry mas was used with the PU basic cocktails.

ii. Preparation of Ag/AgCl Electrodes

All steps were conducted in a dust-free fume hood.

Washing: Ag (99.9%) pins were first soaked in acetone for 10 min. The pins were placed on lint-free paper and the acetone was allowed to evaporate. The pins were then soaked in HCl (37%) for 20 min to get characteristic metallic color of silver. The HCl was neutralized with NaOH and the pins removed. The pins were rinsed four times with deionized water and again allowed to dry on lint-free paper in a dust-free fume hood. The Ag pins were then inserted and glued into hard PVC caps.

Chloridization: Up to 150 pins were chloridized at one time, using an I-tech IT6322 power supply (IT6322 30V/3A*2CH + 5V/3A*1CH) equipped with three channels. Jigs by which 50 pins could be connected to one of the anode channels of the power source were set on top of the base vessels containing 1 M HCl. Platinum counter electrodes placed in all four corners of each chloridizaton vessel were connected to the cathodes of the power supply. The volume of HCl was adjusted so that all of the Ag below the cap of the pin was submerged. A current of 20 ± 3 mA was applied for 1.5 h. The pins were then rinsed, first with tap water and then carefully with deionized water and then dried overnight. All the pins were checked under a microscope to prove uniform coverage of silver by AgCl. The region close to the plastic cap was covered with a thin layer of Loctite 9483 A&B glue to cover any Ag uncovered with AgCl from having any contact with the sample, and to prevent KCl creep through the cap to the electrical connection above.

### 2.3. Electrode and Body Types

**Figure 1:**
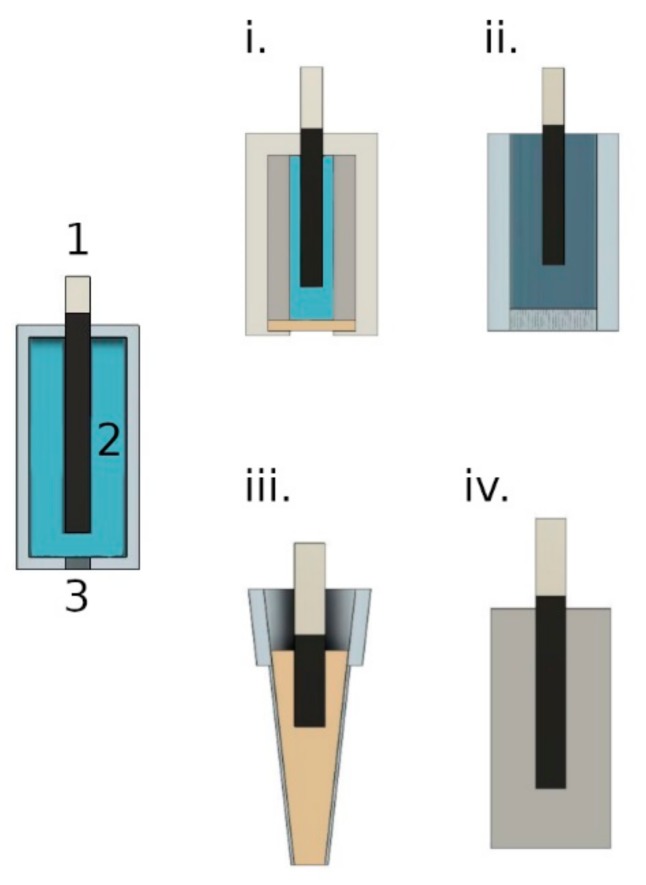
Schematic diagrams of reference electrodes (REs) used. On the left side general scheme of RE, where 1) is the Ag/AgCl electrode, 2) is the internal solution or solid contact, 3) is the membrane or frit. On the right side: (**i**.) RE with QB (PVC) and QB (PU) membranes, (**ii**.) RE with remelted inorganic salts (RKCl), (**iii**.) urea-formaldehyde (UF) resin-based RE, and (**iv**.) RE with PVA membrane. More detailed characterization of (**i**.–**iv**.) type is provided in the text.

Two main classifications of REs were produced. Membrane-based reference electrodes were made of two types (QB (PVC) and QB (PU)), using the same body type. The completely solid-state reference electrodes (SSREs) consisted of three types: remelted KCl reference electrodes, urea-formaldehyde (UF) resin reference electrodes, and poly(vinyl acetate) (PVA). All these RE types are schematically shown in Figure 1.

i. Conventional Liquid Contact Membrane-Based REs

Basic membrane cocktails were prepared as described above. 2 mL of the cocktail was poured into a 24.0 mm glass ring mounted onto a glass plate. The ring was covered with a paper and the THF allowed to evaporate for at least 24 h. A cork borer was used to cut 8.0 mm membrane discs from the master membrane. These 8 mm discs were subsequently mounted into Phillips bodies (see: Figure 1i.)

ii. Molten/Remelted KCl Reference Electrode

The electrode body had to withstand high temperatures and mechanical stress during heating. Quartz glass satisfied these requirements. Two quartz frit porosities were tested, with the denser one giving better performance. Potassium chloride was heated to 500 °C to remove the water. Then the body of the electrode was filled with potassium chloride, and the Ag/AgCl wire (2 mm diameter) was suspended in the KCl. The body was placed in the furnace and heated/cooled in three temperature steps of 25 °C → 500 °C → 820 °C → 750 °C (cooling) with the rates 4, 2, and −0.5 °C/min, respectively. The electrode was kept in the furnace until the temperature returned to 25 °C, about 20 h. The silver wire extending out of the KCl was attached to a Metrohm connector model 6.1241.060. (see: Figure 1ii.)

iii. Urea-Formaldehyde Resin + 25–50% w/w KCl Reference Electrodes

The urea-formaldehyde resin was fabricated as described previously [30]. Numerous trials with different mold types resulted in the conclusion that the UF polymer resin did not adhere well to any mold material at our disposal. The poor adhesion allowed the sample to penetrate up the walls of the electrode in an inconsistent fashion. This resulted in some variation in results depending on the surface area of the UF in contact with the sample and tightness of fit to the mold. Limiting the surface area of UF in contact with solution was quite effective in reducing this problem. The best electrodes were those made in 40–200 µL micropipette tips. Wrapping with teflon tape as recommended by the authors in [30] was somewhat effective in reducing penetration of water, but the best results were nonetheless obtained with the micropipette bodies. (see: Figure 1iii.)

iv. Polyvinyl Acetate (PVA) + 50–70% KCl Reference Electrodes

Potassium chloride and sometimes also lithium chloride were ground in a mortar to get a fine powder. The KCl and LiCl were dried for 30 min at 450–500 °C. The powdered salts were mixed with VA and DMPP. The mixture was placed in a mold to form the RE body with the Ag/AgCl wire affixed in the center of the mold. The mold was sealed and placed on a roller mixer (Stuart SRT6) below a 6 W UV lamp (Vilber Lourmat). The mixture was then mixed for 2 min after which it was irradiated using the UV lamp at 365 nm for 40–50 min (with mixing). After irradiation, the form was left for 2 h to cool down. The mold was removed from the hardened mixture by cutting and peeling it off (if the form was made of plastic) or cracking it off (if the form was made of glass). (see: Figure 1iv.) 

### 2.4. Uniform Testing Protocols

In order to objectively compare the behavior and quality of the investigated reference electrodes of different design, uniform testing protocols were devised and adopted. These protocols were developed to test many factors that can influence the reference electrode behavior.

#### 2.4.1. Stability Testing Protocol

The stability criteria arise in two ways. Firstly, the measuring equipment used to evaluate the electrode stability has its own limitations, which we call the ‘equipment capability criterion’. The noise from the instrumentation under optimal conditions accounts for approximately 0.005 mV of variation. Secondly, the intended use of the electrode also presents its own set of limitations. These types of limitations give rise to what we call the ‘method-based criterion’. If we are measuring in a standard solution after each sample as is often done in clinical analysis, we require only that the drift is small enough that it is negligible in the time frame of two measurements, e.g., 1 min. For instance, if we are measuring Na+ in blood serum, with a normal range of 120–150 mM, with a required coefficient of variation (CV) better than 0.25% [2], the drift could be no more than 0.015 mV/min or 0.87 mV/h as long as it is a stable, regular drift.

On the other hand, as is more typical with, e.g., pH measurements where one would like to measure without a subsequent standard, the reference must remain relatively stable for around one day. In this latter case, we must also specify the intended accuracy of the method over that time span. If we specify an accuracy of 0.1 pH units and assume that the pH electrode itself does not drift, we require a (less stringent) stability of 0.25 mV/h. If we, for example, would rather have a pH test that required calibration only once per week but with 0.2 pH unit accuracy, we would require a (more stringent) stability of approx. 0.05 mV/h.

Potential stability can be described as a property of the electrode to maintain the same potential in certain conditions. It is usually expressed as a standard deviation over a certain period of time. There are two main sources of instability:

Noise, which is a random effect, can be diminished by increasing the number of measurements. It is described as a standard deviation or as a span of the potential. If the noise is expressed as the span, then there is a relation between span and standard deviation as follows: span ≈ 2 x SD. There can be many sources of noise, such as electronic equipment, power supply network, the electrode itself, etc.

Drift, which is a systematic effect, might be corrected (bias) during measurements (considering drift via mathematical formulae or conducting calibrations more frequently). It is usually described as a change (shift) of the potential over time.

In real situations, noise and drift occur simultaneously and can be estimated. However, the drift in most cases is beyond interest due to frequent recalibrations, repeated short measurement times or software corrections, whereas the noise is taken into account to estimate uncertainty.

**Figure 2:**
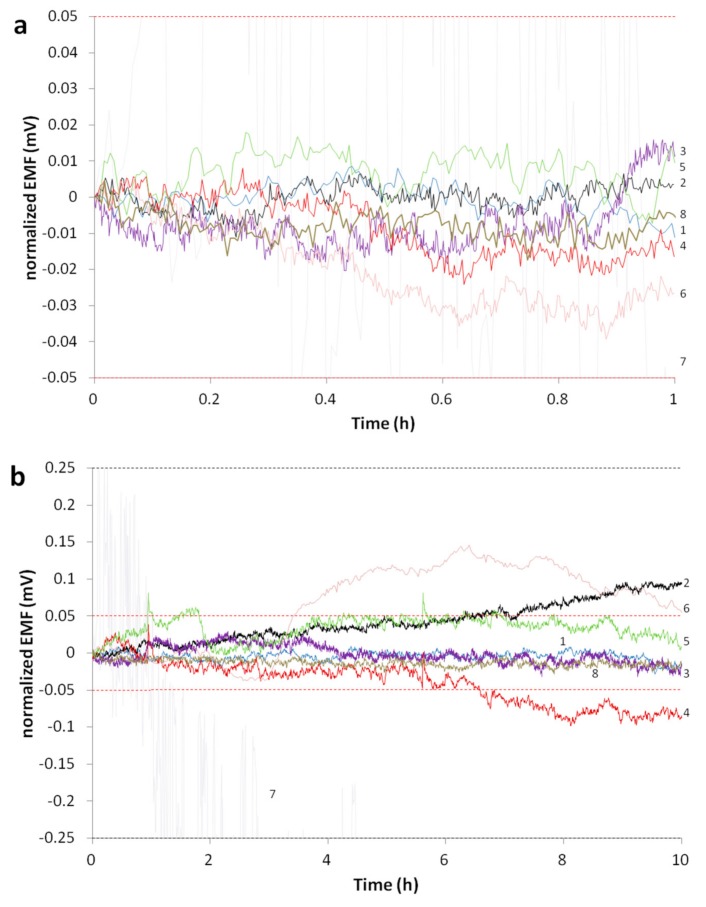

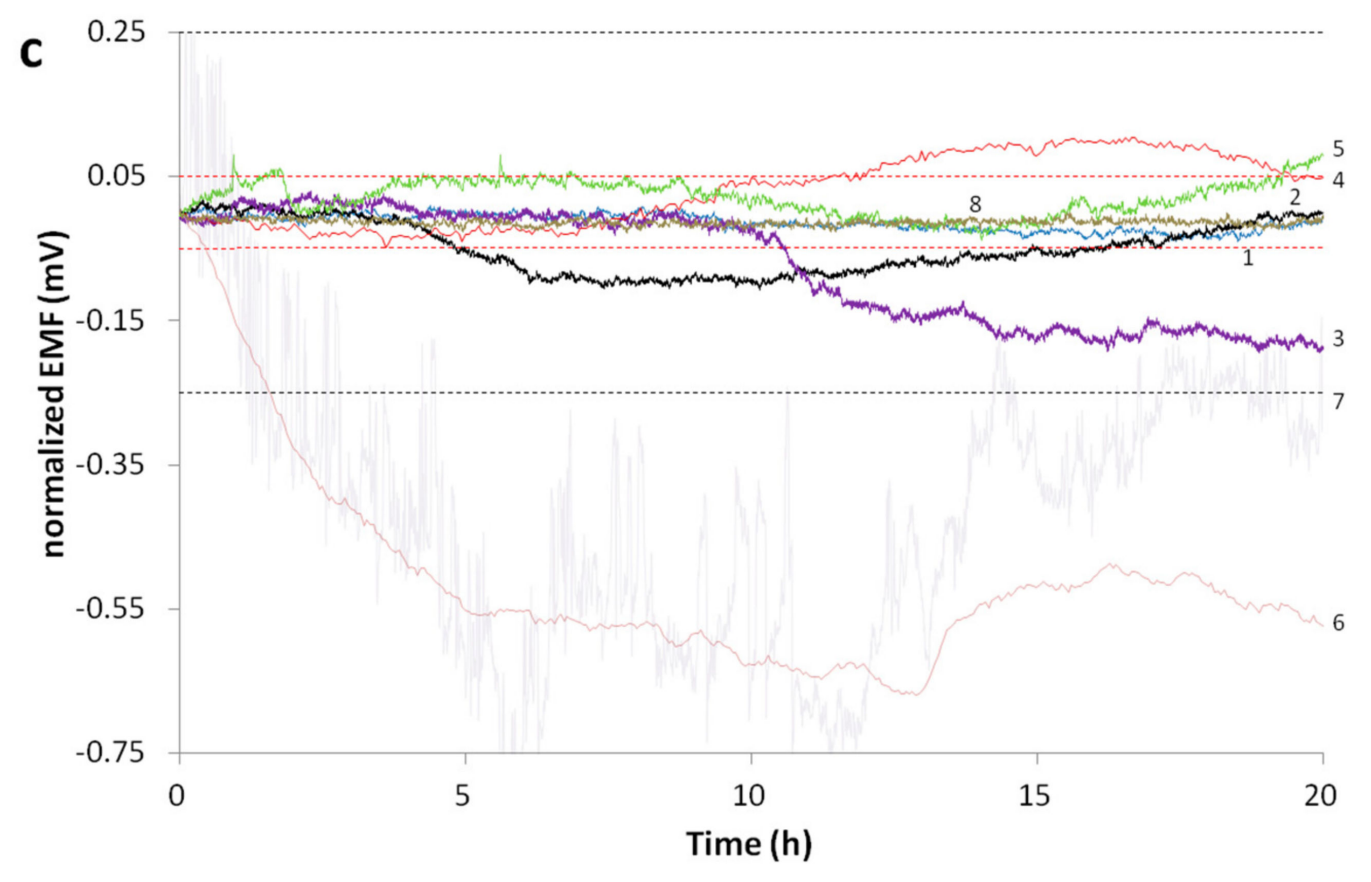
Stability over (**a**) 1 h, (**b**) 10 h, and (**c**) 20 h in 10^−4^ M KCl at 23 °C for 1. QB (PVC), 2. QB (PU), 3. RKCl, 4. PVA, 5. UF, 6. Sureflow, 7. REFEX, 8. Orion Ross Ultra.

Measurements to investigate the long-term stability of the reference electrodes were carried out in 10^−4^ M KCl solution against the commercial ORION 800500U ROSS Ultra D/J RE. The measurements were carried out in a Faraday cage. The data was recorded and collected using a Lawson Labs 16-channel potentiometer. Measurements were taken every 5 seconds and were obtained using the EMF Suite 2.0 software. The standard deviations for measuring-times over 1, 10, and 20 h were calculated. To better compare the results while still being able to visibly see deviations, the potentials were normalized for 1, 10, and 20 h by taking the first point as “0”. The drift was calculated as the linear slope approximation by taking results from the final 30 h. The more stringent tolerance range is represented as two red horizontal dotted lines in the figures. The total potential scale is adjusted to the less stringent criterion, except in Figure 2c, where the range is represented by two black horizontal dotted lines.

#### 2.4.2. pH Testing Protocol

The pH protocol was designed to test the reference electrodes’ stability in a relatively wide range of pH samples and buffers for which the pH is well defined (see: Table 1). A literature search was conducted to establish what samples have traditionally been used to test reference electrodes. The software PHREEQCI was used to check these literature values, and good agreement was found in all cases. The electrodes were measured for 5 min in each sample and the reported results are the average of the final 2 min unless the result was not stable during that time interval.

#### 2.4.3. pH Titration Procedure

Solutions of 0.05 M NaOH and 0.005 M HCl were prepared. The NaOH was standardized using potassium hydrogen phthalate, and then used to titrate 100 ml of 0.005 M HCl in which the tested reference electrodes were immersed. In Table 2 calculated pH values are given. The potentials were recorded using a 16-Channel Lawson Lab potentiometer and EMF Suite 2.0 software. The potential was measured for 5 min after each titration step.

#### 2.4.4. Multi-Solution Testing Protocol (MSP)

This test aimed at studying the effect of the nature and the concentration of the sample electrolyte. The electrode potentials were recorded in the solutions listed below: KCl 3.0 M, deionized water, NaCl 0.01 M, KCl 0.01 M, HCl 0.01 M, deionized water, KCl 3.0 M, NaCl 0.1 M, KCl 0.1 M, NaBr 0.1 M, NaHCO_3_ 0.1 M, KOH 0.001 M, HCl 0.01 M, and KCl 3.0 M, deionized water. The EMF readings were recorded for 5 min in each sample and the reported results are the average of the final 2 min. The electrodes were rinsed with deionized water between samples.

## 3. Results and Discussion

As mentioned above, nowhere has anyone attempted to collect the reference electrodes of interest by the several groups working in this area for comparison testing. Since these references have been designed for different purposes, the tests that have been reported for each type have not been uniform, which makes an independent comparison of them difficult. Such a comparison requires some representative tests to demonstrate which of the references are best for potentiometric tests. Here, we attempt to test in a common fashion the interferences of drift, noise, ionic strength, junction potentials due to ionic mobility differentials, ionic species, pH and buffer species.

### 3.1. Stability

The first issue with any reference electrode is its stability over time. Different applications exert different stresses upon a reference electrode. In our test regime, we chose to use a dilute 10^−4^ M KCl solution as our sample and monitored the reference potential over a period of several days. Such a low concentration was deemed to be enough of a challenge, especially for electrodes from which KCl is presumed to diffuse out into the sample. 

Most of the electrode types we studied performed adequately well to be used for clinical measurements in which an online standard is used for E^0^ correction, and for pH measurements where calibrations are performed daily. The former places no strong demand on reference electrode stability other than that it not be particularly noisy, and all of the references measured could be used for such an application. The latter criterion (pH) was more challenging, but for pH measurements demanding only 0.1 pH unit accuracy, again all of the reference electrodes studied would be acceptable, assuming no extremely large pH changes between samples. If the pH measurement accuracy should be greater, or the calibration interval longer, the Orion Ross Ultra, QB (PVC), and QB (PU) references demonstrated superior stability. Figure 2a–c shows the potentials’ stability of the various reference electrodes graphed together. 

The potentials of the QB (PVC) electrodes: QB30 (PVC)-02, QB30 (PVC)-03, and QB30 (PVC)-11 were quite stable. The standard deviations of QB30 (PVC)-11 for measuring times of 1, 10, and 20 h were 4, 6, and 11 μV respectively, and its drift over the last 30 h of testing was only 1 µV/h. Potential over time for electrode QB30 (PVC)-11 is shown in Figure 2a–c. The QB (PU) electrodes performed similarly well. The QB10 (PU) references showed relatively good stability and drift, but were outperformed in every aspect by the QB25 (PU) for which the lowest drift of all test reference was recorded. The QB25 (PU) references were exceeded only by the Orion Ross Ultra reference electrode in terms of drift. The stability of QB25 (PU)-05 is shown in Figure 2a–c.

The RKCl1 electrode was prepared in a body with a high-density frit (low porosity), whereas RKCl2 electrode with a lower density (higher porosity) frit. The two electrodes showed similar behavior and the same parameters (see Table 3). The standard deviation for measuring times of 1, 10, and 20 h are 7, 42, and 66 μV respectively for the RKCl1 electrode, and 7, 12, and 77 μV respectively for the RKCl2 electrode. Both electrodes drifted 7 μV/h. Long-term time development of the potentials for RKCl1 are shown in Figure 2a–c. The standard potential for the RKCl1 electrode was −221.2 mV and for the RKCl2 it was −221.9 mV against the ORION 800500U ROSS Ultra D/J electrode. These results also showed good reproducibility regarding standard potential as well as stability. 

The UFREs were also stable over a long period of time. All of the UFREs tested had a 20 h SD below 0.2 mV, and in a few cases the SD was even lower than 0.1 mV. All of the UFREs also displayed very small drift below 20 μV/h. These electrodes furthermore showed good reproducibility regarding standard potential. The long time development of the potential of the electrode UF20f is shown in Figure 2.

Our own PVA references also displayed good stability. Only one example, PVA1, is listed in Table 3 since the stability test was not performed in its entirety with any other pieces. Its standard deviations were not high, and it drifted little over time. The commercial REFEX© RE was, however, the least stable of the commercial electrodes over time, and was characterized by significant noise. 

Not surprisingly, the stability of an Orion Ross Ultra against another Orion Ross Ultra was very good. However, strangely an Orion Ross Sureflow RE against the Orion Ross Ultra performed rather poorly, and worse in terms of stability than many of our test references especially in terms of long-term drift. The test was repeated several times with similar results. The experimental references tested here all performed as well or better than the Orion Sureflow double-junction reference and far better than the REFEX®.

### 3.2. pH Response

**Figure 3:**
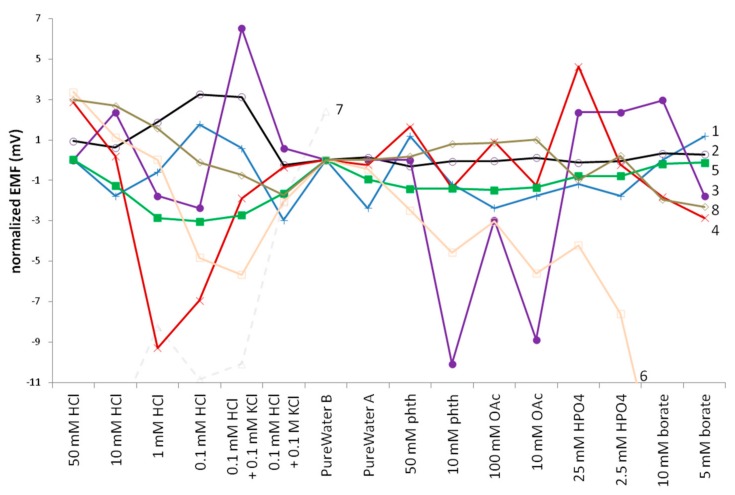
pH titrations with the various reference electrodes tested. 1(+) QB(PVC), 2(o) QB(PU), 3(●) RKCl, 4(×) PVA, 5(■) UF, 6(□) Sureflow, 7(△) REFEX, 8(◇) Orion Ross Ultra.

The pH response of the electrodes was tested in two ways. First, the references were exposed to a series of samples and buffer solutions, to monitor deviations from normality in the potential measured against the ORUDJRE. The buffers used are listed in the experimental section and below the x-axis in Figure 3, and included most of the primary and secondary buffers. Beyond testing merely response to pH, this test also looked at the reaction of the reference electrode to the chemical make-up of the buffering agent and to the sample or buffer ionic strength. The ionic strengths in general were still far higher than would be found in natural/environmental samples, although the 0.1 mM HCl approximates acid rain. 

For most of the experimental electrodes tested, the ionic strength of the buffer had a larger effect than the buffer composition, as evidenced by the relatively large changes in reference response in the measurements in HCl samples. Most telling is the return to nearly normal response upon addition of 0.1 M KCl, which hardly changes the pH. The QB (PU) 25 electrode is particularly impressive in the measurements after the HCl samples, for which the standard deviation of the measured pH over all samples was 0.2 mV. The Orion Sureflow and REFEX REs were very poor, the latter being so bad that the test with it was discontinued after the acid samples.

**Figure 4:**
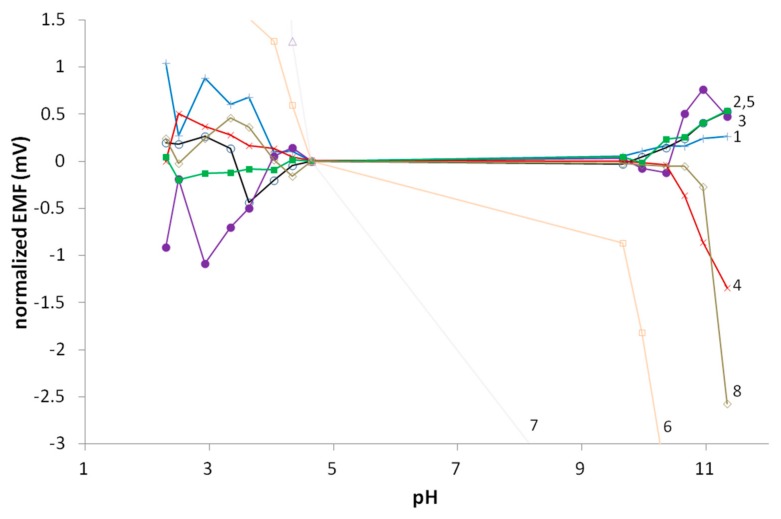
pH titrations with the various reference electrodes tested. 1(+) QB(PVC), 2(o) QB(PU), 3(●) RKCl, 4(×) PVA, 5(■) UF, 6(□) Sureflow, 7(△) REFEX, 8(◇) Orion Ross Ultra.

Second, the electrodes’ responses were monitored during a pH titration. As shown in Figure 4, except for once again the REFEX© and perhaps Orion Sureflow references, all of the REs were useful and stable in the pH range 4–10. These results also suggest that, of the experimental REs, the PVA- and UF resin-based REs are the best choices for measurements below pH 4, while a QB (PVC) RE, QB (PU) RE or UFRE is the best above pH 10. It seems that PVAREs are somewhat more sensitive to pH above 10, probably due to hydrolysis of the matrix polymer. In spite of the slight sensitivity, these REs show good parameters in terms of potential stability and reproducibility. The UFREs show even better behavior, being almost insensitive to pH changes. However, in more acidic or alkaline solution some potential deviation does occur. The commercial Orion Ross Ultra shows very good parameters, although it too displays some sensitivity to pH above 11. Assuming both Orion Ross Ultra reference electrodes are identical, this should not have been the case, so presumably one of our Orion Ross Ultra references was partially clogged. Orion recommended procedures to allow freer flow through the liquid junction to remedy this. The REFEX© which is probably based on PVA or another ester resin is very sensitive to pH values, which leads to a rather high SD as shown in Table 4. It should, however, be pointed out that a deviation of 1 mV is equivalent to an error of just under 0.02 pH units, so apart from the REFEX electrode, any of these would be suitable for fairly accurate pH work. No work has been done to characterize the more complicated interactions of these references with temperature and pH.

### 3.3. Multi-Solution Protocol

**Figure 5:**
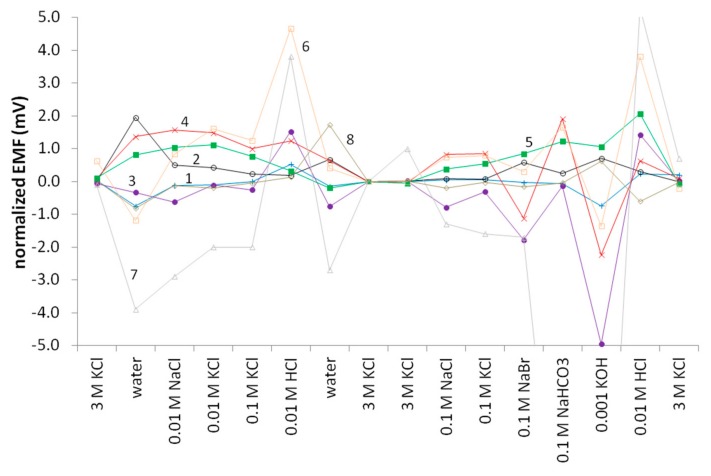
MSP results for the experimental and commercial electrodes tested for this study. 1(+) QB(PVC), 2(o) QB(PU), 3(●) RKCl, 4(×) PVA, 5(■) UF, 6(□) Sureflow, 7(△) REFEX, 8(◇) Orion Ross Ultra.

**Figure 6:**
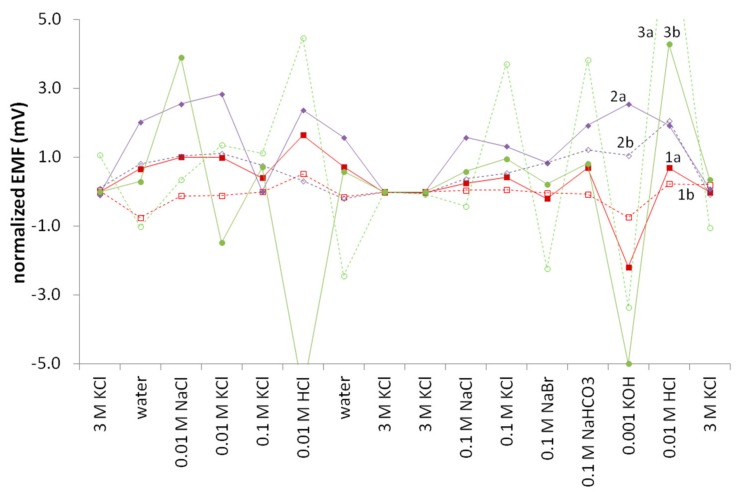
Effect of aging on the 1. QB(PVC), 2. UF, and 3. PVA reference electrodes (**a**) soon after production and (**b**) after three months of semi-regular use.

The 3.0 M KCl, a number of 0.1 and 0.01 M solutions (described in the paragraph 3.4.4 and represented on the x-axis in Figure 5 and Figure 6), and deionized water were chosen to demonstrate the influence of ionic strength. K^+^ and Na^+^ demonstrate the influence of the cation, Cl^−^, Br^−^, and HCO_3_^−^ demonstrate the influence of the anion, while at the same time revealing the effect of disparate ion mobilities. The 0.01 M HCl and 0.001 M KOH show the influence of pH. 

The order of the electrolytes in the series was designed to ensure the minimization of side effects. In particular, if electrodes drift over time, measurements in a number of 0.01 M solutions and 0.1 M solutions one after another reveal the effect of cation or anion with minimal impact from the drift. Measurements in highly alkaline media are relatively seldom, but on the other hand it is always difficult to wash the electrodes and cell after alkaline solutions. This is why the concentration of hydroxide (0.001 M KOH) was lower than that of other electrolytes, and the measurement in KOH was followed by a measurement in 0.01 HCl.

Figure 5 shows the results for the experimental and commercial REs tested. The electrodes incorporating the lipophilic salt QB performed the best, with little differentiation between the PVC or PU supporting membrane. The other types tested also performed well, with the PVA and UF solid REs slightly better than the remelted KCl REs. The Sureflow commercial reference performed slightly more poorly, and the REFEX commercial RE performed very poorly, especially in the HCl and KOH samples as noticed earlier in the pH testing. The remelted KCl and to a lesser extent the PVA electrodes’ performance was influenced by the physical structure and morphology of the electrode bodies. Changing the pH of the sample drastically resulted in generally negative errors in alkaline samples and positive errors in acidic samples because of the glass sinter in the remelted KCl RE bodies and the porosity of the PVA resin. Given enough time (>10–15 min), these errors self-correct but over the short-term errors are recorded. The same effect was reproduced by using a glass sinter body such as that used for the remelted KCl electrodes and filling it with the 20% KCl-UF resin. While its performance generally matched that seen with pipette tip UFREs, the switch from bicarbonate to KOH and then from KOH to HCl resulted in similar errors, as seen with the remelted KCl and PVA REs. Presumably the porosity of the glass sinter was the cause since the same error was not observed with UF micropipette tip REs. The same problem, but greatly exacerbated, is seen with the commercial REFEX electrode.

The age of the electrode also plays an important role, both with traditional types and with any new type, but because of the ratio of the number of electrodes tested to the number of researchers doing the testing, not all of the electrodes were tested uniformly. The QB (PVC) REs were made first, thus allowing for a more thorough investigation of their lifetimes, followed in time order by the solid remelted KCl, PVA, UF electrodes, and finally, the QB (PU) REs. The MSP results for the first three types are shown in Figure 6. The QB (PVC) REs seem to improve somewhat over three months, while the UFREs deteriorated slightly over that same time period. The PVA REs deteriorated more. It is unknown due to the short time frame of the study whether or not these effects are real or due to normal statistical variation.

The problems with UFREs are presumably due to the poor adhesion to every body-type tested except for the glass sinter bodies also used for the remelted KCl REs. It was important to minimize surface area contact between the UF resin and the sample, since a larger surface area in contact with the sample resulted immediately in poor performance. However, even using a micropipette tip as the body, which reduced the surface area to about 0.1 mm^2^, over time it was still evident that the sample or conditioning solution had penetrated up the body walls into the electrode. With most pieces, performance deterioration could already be observed after one month of regular use, although some pieces lasted through the duration of the testing (4 months).

### 3.4. Comments on Manufacturing

Manufacturing of the various types of electrodes was variably challenging. In terms of cost, the remelted KCl electrodes were highest, as the quartz body required for the high-temperature preparation cost EUR 100 alone. The membrane-based reference QB (PVC) and QB (PU) were next in line, since although the membranes are cheap to produce, the bodies into which they are mounted are quite expensive. The solid electrodes were the least expensive, the final price depending on the mass of the electrode, but realistically staying below EUR 1 per electrode.

In terms of complexity of production, the PVA solid references are perhaps most complex due to production requiring distillation, multiple ‘ingredients’, many weighings, and the final polymerization step using various types of initiator, air release agent, accelerator, etc. Problems with electrode hardness, air bubbles, consistent polymerization of the bulk material, etc. must all be contended with. A switch to using a ready polyester (PE) resin would facilitate the procedure, since one avoids distillation and only two ingredients are needed in addition to the commercial hardener, and to a great extent the problems listed above are also avoided. Unfortunately, initial forays with this approach have not yielded equally good performance. It is likely that the commercial polyvinyl ester resins in use contain proprietary ingredients that are not entirely inert with respect to the tested samples. Remelted KCl is simple once one knows how to deal with the high temperatures. Membrane references are extremely simple for anyone having any experience with ion-selective electrodes. 

Tools for reference electrode production are also worth considering. PVA polymerized from the monomer requires distillation apparatus, a mixing system and a UV lamp. UF REs required only a distillation system. Membrane electrodes require almost no hardware, only a cork borer to cut the membranes from the master membrane. Remelted KCl REs require an oven capable of >950 °C.

Polyurethane-based membrane reference electrodes took the longest to make only because the polyurethane takes a few days to dissolve in THF. There is room for improvement in the choice of solvent, but since THF is so ubiquitously used in ISE production, the fact that it can be used with polyurethane is nevertheless a benefit. Otherwise, the PVC-based membrane electrodes require a day for dissolution of the cocktail and another day for membrane casting, and a third day for conditioning. The solid electrodes require some initial hours of preparation and between a few hours to a day for electrode preparation. Conditioning can occur as quickly as in a few minutes, although we normally used a whole day.

The rejection rate of the QB (PVC) references was particularly high, with the percentage rejected between 70–80%. Membranes cut from the same parent membrane and placed into Phillips bodies did not even consistently function identically, indicating either variation in the Phillips bodies or some other uncontrolled variable in the production process. Switching from PVC to PU solved the problem, so the root cause was not pursued. The rejection rate with QB (PU) electrodes was below 20%. With the solid references, rejection rates tended to be lower. The best was perhaps the UF resin-based references in micropipette tips. PVA references did not always work identically. Problems were mostly likely due to centering and depth of the Ag/AgCl wire in the bulk of the solid electrode, air bubbles, and/or hardness after soaking in sample. In a real production process, centering, and depth of the Ag/AgCl pin would be easy to solve. The removal of air bubbles and achieving the desired hardness of the electrode would also not be difficult challenges to overcome with the correct equipment and longer experience. Table 5 collects the production variables for easy comparison.

## 4. Conclusions

The application of a unified set of measurement protocols to multiple reference electrodes allowed us to gain a clearer picture of the shortcomings of the types in question, and to see clearly that these recently reported references are all, in several ways, an improvement over some commercially available but more traditional types of reference electrode. One major shortcoming of this work was the short duration of the testing period which did not give any information concerning the lifetimes of the electrodes. However, the majority of these electrodes are cheap to produce, and even after three months they were mostly superior to two of the commercial electrodes tested. 

It is recommended that, in the future work of other groups involved in the development of reference electrodes, tests be conducted at least similar to those described here. Furthermore, it is critical that the mV range on the y-axis of figures be kept as narrow as possible in order that the reader can clearly differentiate whether or not the reference response is really stable. The goal with a potentiometric reference should be stability to within at most a few mV, and when the y-axis is expanded to hundreds of mV, a change of 10 mV is easily hidden by the sheer scale of the figure. Thus, we recommend normalization of the results as much as possible, indicating the potential against, e.g., Ag/AgCl in some other fashion as deemed necessary.

The solid reference types are of great interest because they offer easy fabrication and possibility of dry storage, fast conditioning time, and low cost. A stable readout and low efflux of KCl into the sample depend on morphology. The flexibility of the latter may be offered by the components of the composite and fabrication patterns. In general, to make optimization of these electrodes most efficient, there is a need for a theoretical support of their operation-module. 

The clear winner of the empirical studies presented, in terms of performance presented, is the QB(PU) reference electrode. Its stability, pH performance and MSP performance were all the best. The results reported create a rich empirical base which will be used by the authors to elucidate the mechanism of the electrochemical performance of the composite heterogeneous membranes. We are a step further to the point where novel 3D composite membrane structures will revolutionize the world of 1D boundary membranes not only in theory, but in electrode fabrication and application scopes.

## Figures and Tables

**Table 1:** pH in different solution used.

Dilute Acids	pH (Pitzer eq) [75,76,77,78]
1. 50 mM HCl	1.38
2. 10 mM HCl	2.05
3. 1 mM HCl	3.02
4. 0.1 mM HCl	4.01
5. 0.1 mM HCl + 0.1 M KCl	4.03
6. 0.1 mM HCl + 1 M KCl	4.09
**Buffers & Dilute Buffers**	-
7. Orion PureWater buffer A	6.97
8. Orion PureWater buffer B	4.10
9. 50 mM potassium hydrogen phthalate	4.01
10. 10 mM potassium hydrogen phthalate	4.12
11. 100 mM HOAc/100 mM NaOAc	4.65
12. 10 mM HOAc/10 mM NaOAc	4.71
13. 25 mM KH_2_PO_4_/25 mM Na_2_HPO_4_	6.88
14. 2.5 mM KH_2_PO_4_/2.5 mM Na_2_HPO_4_	7.06
15. 10 mM disodium tetraborate	9.18 [79,80] ^☨^
16. 5 mM disodium tetraborate	9.20 [13], 9.21 [5], 9.19 [79]

^☨^ PHREEQCI’s database did not contain the relevant information for boric acid/borate, so values were found in the literature.

**Table 2:** Calculated pH for pH titration.

NaOH mL	0	3.5	7.5	9	9.5	9.8	9.9	9.95	10.1	10.2	10.5	11	12	15
pH (calc)	2.30	2.50	2.93	3.34	3.64	4.04	4.34	4.64	9.66	9.96	10.35	10.65	10.95	11.34

**Table 3:** Stability data for the REs tested.

Electrode	Stability * [μV]			Drift ** [μV/h]
	1 h SD	10 h SD	20 h SD	
QB30(PVC)-03	35	62	124	3
QB30(PVC)-11	4	6	11	1
QB10(PU)-1	5	29	94	23
QB10(PU)-3	8	28	163	19
QB25(PU)-3	3	35	42	0.8
QB25(PU)-5	3	27	36	0.4
RKCl1	7	12	66	7
RKCl2	7	12	77	7
PVA1	10	29	160	21
UF20e	16	34	42	16
UF20f	15	15	24	9
Orion Ross Ultra	3	4	4	0.02 ≈ 0
Orion Ross Sureflow	122	57	140	95
REFEX©	99	224	195	10

* Stability was calculated as the standard deviation for measuring times 1, 10, and 20 h. ** Drift was calculated as the linear slope approximation from the final 30 h of measurement.

**Table 4:** Parameters of the tested REs in the pH titration.

RE	QB (PVC) 30-11	QB (PU) 25-3	QB (PU) 25-5	RKCl	PVA1	PVA6	UF20e	UF20f	Orion Sureflow	Orion Ross Ultra	REFEX
SD [mV]	0.33	0.24	0.44	0.53	0.55	0.26	0.22	0.21	3.8	0.73	12

**Table 5:** Production variables for the various reference electrode types.

RE Type	Cost Est. *	Complexity/Tooling	Production Time Total (d)/Work (h) ^☨^	Rejection Rate	Issues	Sum
QB30(PVC)	200 € ^§^	medium	3/2	80%	rejection	−−
QB25(PU)	200 € ^§^	medium	5/2	20%	time to obtain	++
RKCl	120 €	high	1/8	0%	cost	−
PVA	0.37 €	high	1/2	10%	bubbles, hardness, pin placement	+
UF resin	0.43 €	low	1/1	10%	body wall adhesion	+

* Ag/AgCl pin price ignored in all cases; ^§^ Phillips body 99.5% of the total price; use of a simplified plastic body drops the price by an order of magnitude.^☨^ work time to produce 10 electrodes.

## Abbreviations

CP	Conducting Polymer
DMPP	2,2-Dimethoxy-2-Phenylacetophenone
ISE	Ion Selective Electrode
MSP	Multi Solution Protocol
ORUDJRE	Orion Ross Ultra Double Junction Reference Electrode
QB	tetrabutylammonium tetrabutylborate
QB (PVC) RE	Reference Electrode with QB/PVC Membranes
Example: QB30(PVC)-01 means the membrane No 01 with QB content 30% w/w dispersed in PVC	
QB (PU) RE	Reference Electrode with QB/PU Membranes
QRE	Quasi Reference Electrode
PE	Polyester
PEDOT	Poly(3,4-Ethylenedioxythiophene)
PMPy	Poly(1-Methylpyrrole)
PTFE	Poly(Tetrafluoroethylene)
PU	Polyurethane
PVA	Poly(Vinyl Acetate)
PVC	Poly(Vinyl Chloride)
RE	Reference Electrode
RKCl	Remelted KCl
SCs	Solid Contacts
SCRE	Solid Contact Reference Electrode
SD	Standard Deviation
THF	Tetrahydrofuran
UF	Urea Formaldehyde Resin
UFREs	Urea Formaldehyde Resin based Reference Electrode
UV	Ultraviolet Lamp
VA	Vinyl Acetate
